# Homologous recombination deficiency in diverse cancer types and its correlation with platinum chemotherapy efficiency in ovarian cancer

**DOI:** 10.1186/s12885-022-09602-4

**Published:** 2022-05-16

**Authors:** Hao Wen, Zheng Feng, Yutong Ma, Rui Liu, Qiuxiang Ou, Qinhao Guo, Yi Shen, Xue Wu, Yang Shao, Hua Bao, Xiaohua Wu

**Affiliations:** 1 Department of Gynecologic Oncology, Fudan University Shanghai Cancer Center, 270 Dongan Road, Shanghai, 200032 China; 2 Department of Oncology, Shanghai Medical College, Fudan University, 130 Dongan Road, Shanghai, 200032 China; 3 Geneseeq Research Institute, Nanjing Geneseeq Technology Inc, No. 128 Huakang Road, Pukou District, Nanjing, Jiangsu, 210000 China; 4 R&D, Nanjing Geneseeq Technology Inc, No. 128 Huakang Road, Pukou District, Nanjing, Jiangsu, 210000 China; 5 School of Public Health, Nanjing Medical University, Jiangning District, 101 Longmian Avenue, Nanjing, Jiangsu, 211166 China

**Keywords:** Homologous recombination deficiency, *BRCA1/2*, Platinum chemotherapy, NGS

## Abstract

**Background:**

Homologous recombination deficiency (HRD) is a molecular biomarker for administrating PARP inhibitor (PARPi) or platinum-based (Pt) chemotherapy. The most well-studied mechanism of causing HRD is pathogenic *BRCA1/2* mutations, while HRD phenotype is also present in patients without *BRCA1/2* alterations, suggesting other unknown factors.

**Methods:**

The targeted next-generation sequencing (GeneseeqPrime® HRD) was used to evaluate the HRD scores of 199 patients (Cohort I). In Cohort II, a total of 85 Pt-chemotherapy-treated high-grade serous ovarian cancer (HGSOC) patients were included for investigating the role of HRD score in predicting treatment efficacy. The concurrent genomic features analyzed along HRD score evaluation were studied in a third cohort with 416 solid tumor patients (Cohort III).

**Results:**

An HRD score ≥ 38 was predefined as HRD-positive by analyzing Cohort I (range: 0–107). Over 95% of the *BRCA1/2*-deficient cases of Cohort I were HRD-positive under this threshold. In Cohort II, Pt-sensitive patients have significantly higher HRD scores than Pt-resistant patients (median: 54 vs. 34, *p* = 0.031) and a significantly longer PFS was observed in HRD-positive patients (median: 548 vs. 343 days, *p* = 0.003). Furthermore, *TP53*, *NCOR1*, and *PTK2* alterations were enriched in HRD-positive patients. In Cohort III, impaired homologous recombination repair pathway was more frequently observed in HRD-positive patients without *BRCA1/2* pathogenic mutations. The alteration enrichment of *TP53*, *NCOR1*, and *PTK2* observed in Cohort II was also validated by the ovarian subgroup in Cohort III.

**Conclusions:**

Using an in-house HRD evaluation method, our findings show that overall HRR gene mutations account for a significant part of HRD in the absence of *BRCA1/2* aberrations, and suggest that HRD positive status might be a predictive biomarker of Pt-chemotherapy.

**Supplementary Information:**

The online version contains supplementary material available at 10.1186/s12885-022-09602-4.

## Background

Genome integrity can be easily affected by environmental and cellular factors which then leads to genome instability and causes tumorigenesis. A versatile and comprehensive DNA damage repair (DDR) network is essential against these endogenous and exogenous insults. Multiple DDR pathways have been uncovered which are responsible for diverse types and magnitude of damage. For instance, mismatch repair (MMR), nucleotide excision repair (NER), and base excision repair (BER) machinery are able to restore DNA single-strand breaks (SSBs) [1–3]. While DNA double-strand breaks (DSBs) can be repaired by either homologous recombination repair (HRR) or non-homologous end joining (NHEJ) [4].

*BRCA1/2* are two key players of the HRR pathway which uses the sister chromatid as the template to complete an error-free DNA repair. Both deleterious mutation and promoter methylation of *BRCA1/2* could cause homologous recombination deficiency (HRD) and genomic instability [5]. Several genomic scars including loss of heterozygosity (LOH) [6], telomeric allelic imbalance (TAI) [7], and large-scale state transitions (LST) [8] were found to be associated with HRD and *BRCA1/2* deficiency. *BRCA1/2* pathogenic variants increase the risk of multiple cancers including breast, ovarian, prostate, pancreatic, and uterine cancers which are identified as *BRCA-*associated cancers [9]. Furthermore, a series of HRR genes including but not limited to *ATM, PALB2,* and *RAD51C* might also lead to similar molecular characteristics termed as “BRCAness” in cells lacking *BRCA1/2* pathogenic mutations [10].

HRR has become a therapeutic target in *BRCA-*associated cancers. Both germline and somatic *BRCA1/2* pathogenic variants are biomarkers for administrating poly ADP ribose polymerase inhibitors (PARPi) [11]. Beyond *BRCA1/2,* HRD score calculated by the sum of LOH, TAI, LST scores was also identified as a biomarker since patients with high HRD scores were reported to respond well to PARPi treatment in breast and ovarian cancer [12, 13]. Moreover, HRD score could identify good responders to neoadjuvant platinum chemotherapy in triple-negative breast cancer even including *BRCA1/2* non-mutated patients [14]. HRD has also been identified as a biomarker for platinum monotherapy in ovarian cancer with both canonical and exploratory HRD score thresholds (42 vs. 33) [15]. However, the mutational landscape of patients with elevated levels of HRD, particularly in *BRCA1/2-* sufficient patients, is largely unclear. Thus, we developed a next generation sequencing panel-based HRD score evaluation pipeline and validated HRD threshold with platinum chemotherapy efficacy in ovarian cancer followed by a comprehensive analysis of the mutational profiles in over 400 tumor tissue samples with diverse cancer types.

## Methods

### Patients

Tumor tissue samples and paired blood samples were collected from a total of 700 patients with diverse cancer types. All samples underwent GeneseeqPrime HRD panel targeting 425 cancer-relevant genes and over 12,000 single nucleotide polymorphisms (SNPs) in a Clinical Laboratory Improvement Amendments-certified, College of American Pathologists-accredited, and International Organisation for Standardisation (ISO15189)-certified laboratory (Nanjing Geneseeq Technology, Jiangsu, China). This study was approved by the ethics committee of Fudan University Shanghai Cancer Center, China (Approval No. 2007221–5). All participants provided written informed consent prior to sample collection. The level of residual tumor after surgery in Cohort II was evaluated by experienced physicians (R0: complete resection of all visible disease; R1: remaining small volume disease ≤ 1 cm; R2, remaining disease > 1 cm [16]).

### DNA extraction and sequencing

Genomic DNA extraction and purification were performed with the DNeasy Blood & Tissue Kit (Qiagen) from white blood cells or the QIAamp DNA FFPE Tissue Kit (Qiagen) from formalin-fixed paraffin-embedded (FFPE) samples, which was then quantified by a Qubit Fluorometer (Life Technologies) with the dsDNA HS Assay Kit. Sequencing libraries were prepared using the KAPA Hyper Prep Kit (KAPA Biosystems), As described previously [17], the indexed DNA libraries for sequencing were prepared (KAPA Hyper Prep Kit, KAPA Biosystems) and captured by probe-based hybridization, which targeted over 400 cancer-related genes and over 12,000 SNPs that evenly distributed throughout the whole genome. The Illumina HiSeq4000 platform was used for DNA sequencing.

### Sequencing data processing

The analysis process of sequencing data was briefly described here. The sequencing reads whose quality less than 15 or N bases were removed using Trimmomatic [18] and the remaining reads were mapped to the reference (human reference genome, hg19) by the Burrows-Wheeler Aligner (https://github.com/lh3/bwa/tree/master/bwakit). The removal of PCR duplicates was done by Picard (https://broadinstitute.github.io/picard/), followed by local realignments with the Genome Analysis Toolkit (GATK) (https://software.broadinstitute.org/gatk/). The tools for somatic single nucleotide variations and indels analysis were VarScan2 [19] and Mutect2. The cutoff of mutation detection was 2% of allele frequency and at least three mutant reads. Based on the 1000 Genomes Project or the Exome Aggregation Consortium (ExAC) 65,000 exomes database, common SNPs with more than 1% of population frequency were excluded. A normal pool of 500 whole blood samples was generated for further mutation filtering to remove any recurrent artifacts. Gene-level copy number alterations (CNAs) were detected using CNVkit (https://cnvkit.readthedocs.io) The cutoff of log2 ratio was set at ± 1 for copy number changes (corresponding to gene amplification and gene deletion).

### HRD score calculation pipeline

Tumor genome-wide allele-specific segment-level copy number profiles are analyzed by its matched normal sample (Pair Model) or a pool of 400 normal samples (Single Model) using PureCN R package (https://github.com/lima1/PureCN), producing allele-specific copy number estimates (per segment total copy number (tCN) and minor copy number (mCN)). HRD score is calculated based on the genome-wide allele-specific copy number result and composed of three parts: 1) Loss of heterozygosity (LOH): the number of segments with ≥ 15 Mb length (but not cover the whole chromosome), mCN = 0, and tCN > 0 [6]; 2) telomeric allelic imbalance (TAI): the number of segments with allelic imbalances (mCN ! = tCN—mCN) extend to the telomeric end of a chromosome [7]; 3) large-scale state transitions (LST), number of chromosomal breaks between adjacent segments of at least 10 Mb, with a distance between them not larger than 3 Mb [8].

### BRCA status classification and homologous recombination repair (HRR) gene pathogenicity

Somatic and germline *BRCA1/2* mutations were detected. Nonsense, frameshift, and pathogenic/likely pathogenic alterations defined by the American College of Medical Genetics and Genomics (ACMG) guideline were identified as pathogenic alterations. The *BRCA*-intact group was comprised of samples without any pathogenic *BRCA1/2* alterations. Biallelic pathogenic alterations, monoallelic pathogenic alteration accompanied by heterozygous deletion, homologous gene deletion, and large genome rearrangement were classified as the *BRCA*-deficient group. The rest samples with only monoallelic pathogenicity were grouped as well. Both *BRCA*-intact and monoallelic pathogenic groups were *BRCA* non-deficient*.* A total of 25 HRR genes (Supplementary Table S1) were covered by the next generation sequencing (NGS) panel whose nonsense, frameshift, and any mutations defined as pathogenic/likely pathogenic in the ClinVar database were identified as pathogenic alterations in this study.

### Statistical analysis and survival analysis

Data were analyzed using R 3.6.3. Categorical variables between groups were compared using χ2 or Fisher’s exact test. Continuous variables between groups were analyzed using the Wilcoxon test. Kaplan–Meier method was used to determine median progression-free survival (PFS) and the significance of survival analysis was determined by the log-rank test. Prognostic indicators including clinical characteristics and HRD score were analyzed using the multivariable Cox proportional hazards model.

## Results

### HRD score evaluation pipeline establishment

**Fig. 1:**
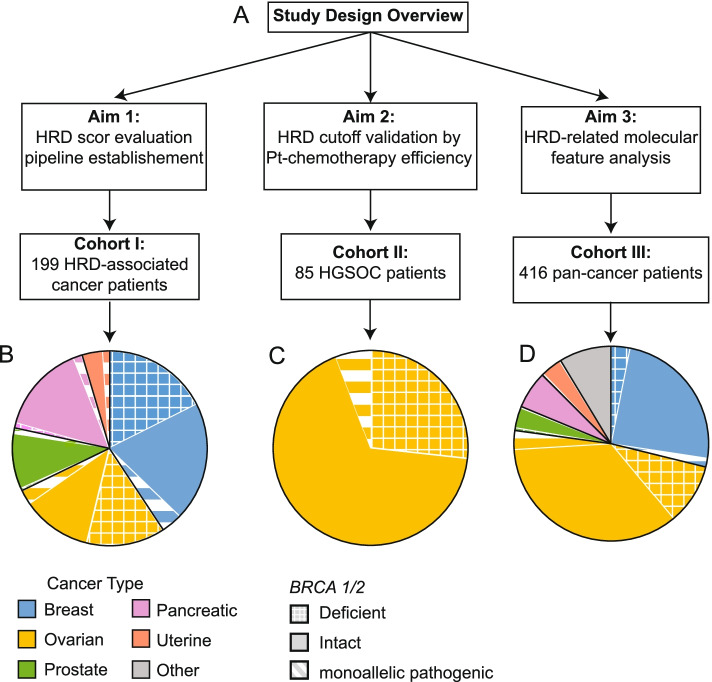
The overview of study design and the composition of the three cohorts. **A** The three aims of this study and the involved cohorts are briefly described. **B**-**C** The cancer type distribution of the three cohorts is labeled by different colors as shown in the legend. The *BRCA* status was classified into three subgroups: deficient, intact, and monoallelic pathogenic as described in the Method section

**Fig. 2:**
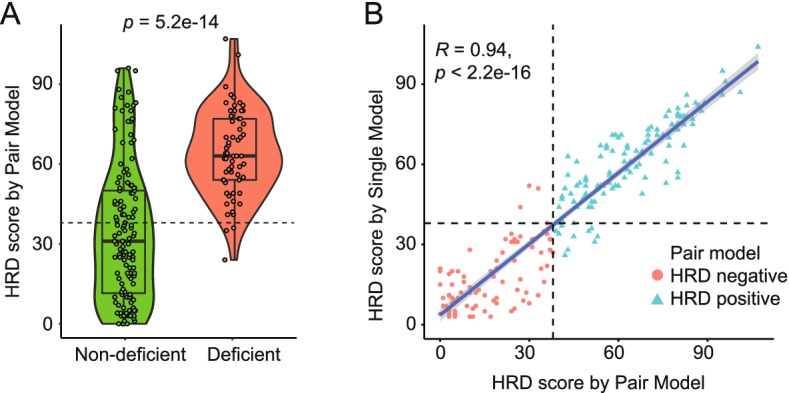
HRD score evaluation in Cohort I and the score comparison between the Pair and Single Model. **A** The HRD scores evaluated by the Pair Model of Cohort I are shown and grouped based on *BRCA* status (deficient vs. non-deficient: intact + monoallelic pathogenic). The dash line (HRD = 38) represents the threshold of positive-HRD that accounts for over 95% (61/64) of the BRCA-deficient samples. **B** The HRD scores evaluated by Pair and Single Model of each sample are shown on the x- and y-axis, respectively in the dot plot. Positive and negative HRD status using HRD ≥ 38 as the cutoff (dash lines) are labeled by green triangles and red circles, respectively, according to Pair Model evaluation

To establish an HRD score evaluation pipeline (Fig. 1A), a cohort of 199 patients diagnosed with HRD-associated cancer types including breast (41%), ovarian (27%), prostate (11%), pancreatic (17%), and uterine (4%) cancers (Fig. 1B) were enrolled as Cohort I (Supplementary Table S2). *BRCA*-deficient samples were mostly identified in breast and ovarian cancers. Matched tumor tissue and blood samples both underwent targeted NGS to calculate HRD score (Pair Model, see Methods section for details). As expected, the HRD scores of *BRCA*-deficient samples were significantly higher than non-deficient ones (median 63 vs. 31, *p* = 5.2e-14, Fig. 2A). HRD score ≥ 38 was defined as HRD-positive that accounted for approximately 95% of the *BRCA*-deficient samples. High-level accordance of HRD scores calculated based on Pair Model and Single Model was shown in Fig. 2B (*R* = 0.94). Thus, the threshold of 38 was also applied to the Sing Model for positive HRD identification.

Furthermore, a subset of 49 patients from Cohort I also underwent whole-genome sequencing (WGS), whose WGS-based HRD scores were compared to those evaluated by the Pair or Single Models. As shown in Supplementary Figure S1, panel-based HRD score was highly correlated with WGS-based HRD score in either Pair or Single stream (*R* = 0.97, *p* < 2.2e-16). The HRD status defined by the HRD score of  ≥ 38 also showed high concordance between panel-based and WGS-based results (Cohen’s kappa = 0.81, Supplementary Table S3: Pair Model vs. WGS and Table S4: Single Model vs. WGS). These results demonstrated that our panel-based HRD pipeline showed high performance in comparison to the WGS-based approach, which represents a gold standard in pipeline benchmarking.

### HRD validation with platinum chemotherapy efficacy

**Fig. 3:**
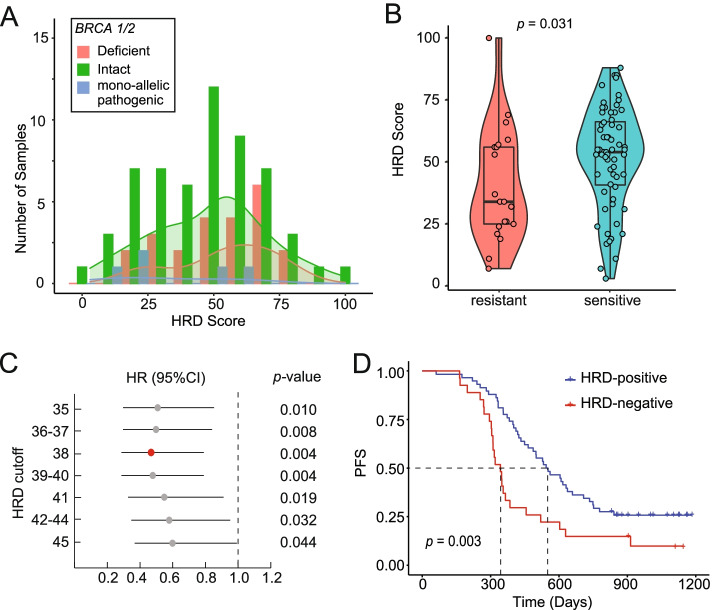
HRD score distribution and survival analysis of Cohort II. **A** The distribution of HRD scores in each *BRCA* status is shown by the histogram plot labeled by different colors as shown in the legend. **B** The HRD scores of platinum chemotherapy-resistant and sensitive patients are shown. **C** Cox regression analysis was performed to predict prognosis when using different HRD scores (35–45) as the HRD-positive cutoff. Hazard ratio (HR) and 95% confidence interval (CI) are shown by the forest plot. **D** The Kaplan–Meier progression-free survival (PFS) curves of HRD-positive and HRD-negative patients are colored in blue and red, respectively

Cohort II, a total of 85 high-grade serous ovarian cancer (HGSOC) patients, underwent surgical removal and then received platinum-based chemotherapy. The surgery-resected tumor samples were analyzed by the HRD Single Model due to the absence of matched blood samples. Most patients were diagnosed as Stage III (89.6%) with a median age of 54 ranging from 37–83 (Table 1). Nearly half of them achieved complete surgery remission (R0, 49.4%, Table 1, Supplementary Table S5). Platinum (Pt)-sensitive patients with a platinum-free interval (PFI) of over six months accounted for three-quarters of the entire cohort (64/85, 75.3%). 67.1% (57/85) of patients were classified into the *BRCA*-intact subgroup and the rest were detected with pathogenic *BRCA1/2* mutations (*BRCA*-deficient, 27.1%; monoallelic pathogenic, 5.8%, Fig. 1C). The distribution of HRD scores was shown in Fig. 3A and the Pt-sensitive patients showed significantly higher HRD scores than Pt-resistant ones (median: 54 vs. 34, *p* = 0.031, Fig. 3B).

**Table 1:** Clinical characteristics of Cohort II

Characteristics	Cohort II: N (%)
Age: median (range)	54 (37–83)
Stage:	
III	76 (89.6)
IV	9 (10.6)
Residual tumor:	
R0	42 (49.4)
R1	37 (43.5)
R2	6 (7.1)
Pt-chemo response	
Sensitive	64 (75.3)
Resistant	21 (24.7)
*BRCA* status:	
Intact	57 (67.1)
Deficient	23 (27.1)
Monoallelic pathogenic	5 (5.8)

The progression-free survival (PFS) data were analyzed based on *BRCA* and HRD status. Firstly, to validate the HRD cutoff based on survival data, HRD scores between 35 and 45 were analyzed using Cox regression to investigate its role in predicting prognosis. As shown in Fig. 3C, the score of 38 showed the smallest hazard ratio (HR, 0.47) and *p-*value (0.004) which was consistent with the cutoff of Cohort I. A significantly longer PFS was observed in HRD-positive patients when using 38 as the cutoff (median: 548 vs. 343 d, *p* = 0.003, Fig. 3D). Even for *BRCA*-intact patients, positive HRD also indicated better prognosis (median: 556 vs. 349 d, *p* = 0.019, Supplementary Figure S2A). While due to the limited number of patients carrying any pathogenic *BRCA1/2* mutations, only a trend of longer PFS was observed in HRD-positive patients but not statistically significant (median: 522 vs. 287 d, *p* = 0.073, Supplementary Figure S2B). However, the pathogenic *BRCA1/2* mutations were not associated with better survival in this cohort (median: 456 vs. 476 d, *p* = 0.62, Supplementary Figure S2C). When combining two clinical factors, cancer stage, and residual tumor after surgery, we performed a multivariate Cox regression analysis and showed that HRD status was the only significant prognostic factor with an HR of 0.463 (Table 2).

**Table 2:** Multivariate Cox regression analysis of Cohort II

Factor	HR	95% CI	*p*-value
HRD (positive vs. negative)	0.463	0.265–0.810	0.0069
Stage (IV vs. III)	1.322	0.588–2.972	0.4991
Residual Tumor:			
R0	-	-	-
R1	1.002	0.572–1.754	0.994
R2	1.082	0.413–2.833	0.873

**Fig. 4:**
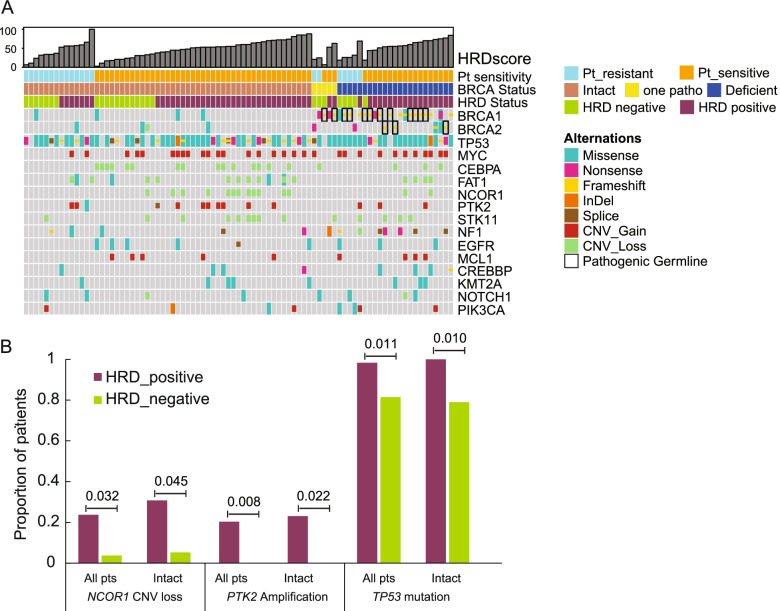
The concurrent alteration analysis of Cohort II. **A** The concurrent alterations detected by the HRD evaluation pipeline were shown by the oncoprint plot. Mutational types and sample features are shown in the legend. The top frequently detected alterations are included here. **B** The proportions of *NCOR1* copy number loss, *PTK2* amplification, and *TP53* mutation are shown by the bar plot where HRD-positive and negative patients are colored differently. The frequency comparison was done in all patients in Cohort II and BRCA-intact subgroup. The *p-*values are shown as well

Concurrent alteration analysis revealed the highest frequency of *TP53* mutations in Cohort II (79/85, 92.9%, Fig. 4A) which was more enriched in HRD-positive patients (98.3% vs. 81.5%, *p* = 0.011). Furthermore, *NCOR1* copy number loss and *PTK2* amplification also showed significant enrichment in HRD-positive patients and the latter one was exclusive in the positive HRD subgroup (Fig. 4B). The complete mutation list of all samples is provided in Supplementary Table S6.

### HRD score distribution and its correlation with other alterations in a pan-cancer cohort

**Fig. 5:**
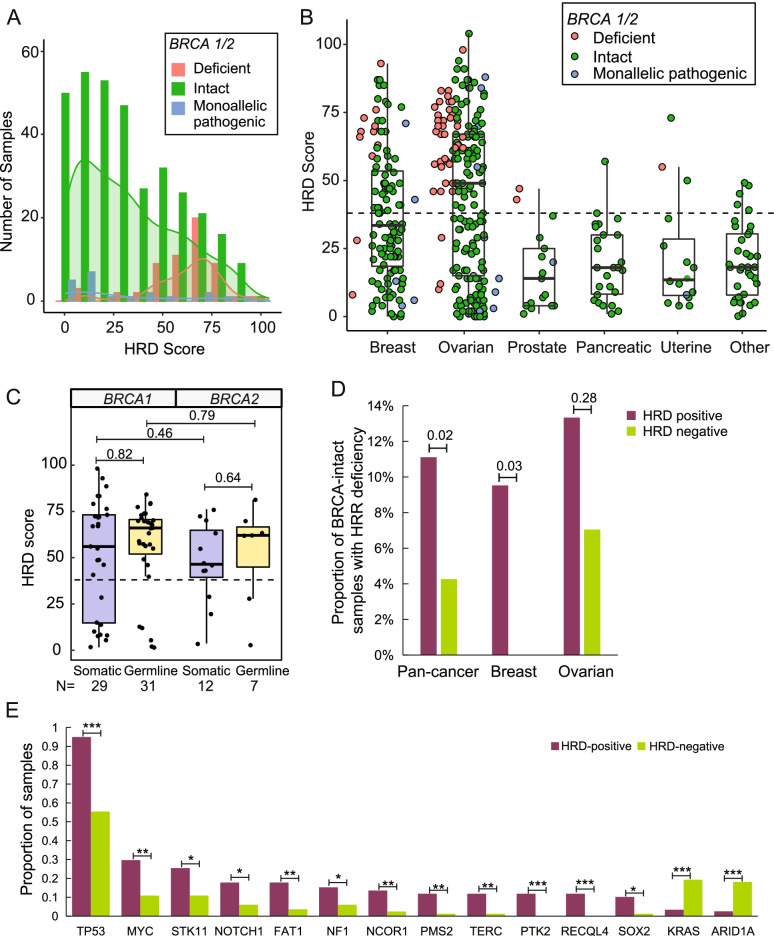
HRD score and molecular feature analysis of Cohort III. **A** The distribution of HRD scores in each *BRCA* status is shown by the histogram plot labeled by different colors as shown in the legend. **B** The HRD scores of each cancer type are shown by the dot/boxplot where BRCA-status are labeled by different colors as shown in the legend. **C** The HRD scores of samples with pathogenic *BRCA1/2* mutations (both deficient and monoallelic pathogenic samples) are shown by the dot/boxplot and classified into somatic pathogenicity and germline pathogenicity. **D** The proportion of BRCA-intact patients with gene deficiency in the HRR pathway are shown in the bar plot and colored based on HRD positivity. The *p-*values of pan-cancer (all BRCA-intact patients in Cohort III), breast, and ovarian cancer patients are shown. **E** The concurrent mutation analysis shows the significantly differently enriched gene alterations in HRD-positive and HRD-negative ovarian patients. *, *p* < 0.05; **, *p* < 0.01; ***, *p* < 0.001

To investigate the molecular features of HRD in a real-world database, the clinical information and HRD test results of a total of 416 patients diagnosed with HRD-related cancers (breast 29%, ovarian 48%, prostate 4%, pancreatic 6%, uterine 4%) and other cancer types (9%) were retrospectively reviewed (Fig. 1D, Supplementary Table S2 & S7). Targeted NGS revealed 58 patients were identified as *BRCA*-deficient (42 ovarian, 13 breast, 2 prostate, and 1 uterine) with a median HRD score of 66.5 as shown in Fig. 5A. Despite the higher frequency of *BRCA*-deficient patients in breast and ovarian cancers, HRD score was significantly higher in these two cancer types even for *BRCA*-intact samples (Fig. 5B).

The correlation between somatic/germline *BRCA1/2* pathogenic alteration and HRD score was analyzed. A total of 79 patients carried somatic or germline *BRCA1/2* pathogenic alterations, 58 of them were *BRCA*-deficient and the rest were monoallelic pathogenicity. As shown in Fig. 5C, more *BRCA1* pathogenicity was detected than *BRCA2* (60 vs. 19) and the HRD scores of *BRCA1-*pathogenic samples were slightly higher than *BRCA2* but not statistically significant. Meanwhile, germline pathogenicity also led to a higher HRD score than somatic ones in both *BRCA1-* and *BRCA2-*pathogenic patients.

For the *BRCA*-intact patients, we investigated the deficiency of other HRR pathway genes. The median HRD score of the 337 *BRCA*-intact samples was 28 ranging from 0 to 104. Over one-third of them (126/337) were defined as HRD-positive mainly in breast (42/126) and ovarian (75/126) cancer. A higher percentage of gene deficiency in the HRR pathway was observed in the HRD-positive subgroup than HRD-negative subgroup not only in all *BRCA*-intact patients (11% vs. 4%, *p* = 0.02) but also in breast (9.5% vs. 0%, *p* = 0.03) and ovarian (13% vs. 7%, *p* = 0.28) cancers (Fig. 5D).

Concurrent gene alteration analysis in ovarian cancer from Cohort III also revealed *TP53* as the most frequently mutated gene which was significantly enriched in the HRD-positive subgroup (Fig. 5E). Besides, the higher frequencies of *NCOR1* and *PTK2* alterations were also observed in HRD-positive ovarian patients which were consistent with the findings in Cohort II (Fig. 4B). All gene alterations with an overall frequency of over 5% in the ovarian subgroup and significantly different enrichment (*p* ≤ 0.05) in HRD positive/negative patients were shown in Fig. 5E. To be noted, only *KRAS* and *ARID1A* mutations were more commonly detected in HRD-negative ovarian patients. In addition, we also investigated the mutational landscape in the entire Cohort III with multiple cancer types. As ovarian cancer accounted for nearly half of Cohort III, most of the genes with significantly different frequencies in HRD positive/negative patients with diverse cancer types (Supplementary Figure S3) were the ones observed in the ovarian subgroup analysis (Fig. 5E). But *PIK3CA* alterations were more enriched in HRD-negative pan-cancer patients but not in ovarian cancer.

## Discussion

In this study, we established two comparable HRD score evaluation pipelines with or without matched normal blood control samples (Pair Model vs. Single Model). In the clinical setting, a sizable population was absent of matched normal samples when detecting HRD for administrating treatment decisions. Thus, the Single Model could be a valuable and efficient tool in the real world. In a previous study on triple-negative breast cancer, HRD ≥ 42 was defined as the cutoff of positive HRD with 95% sensitivity to detect *BRCA*-deficient samples [14]. Based on the Pair Model results of Cohort I, any cutoff between 36 and 41 could lead to the over 95% sensitivity for identifying *BRCA*-deficient samples due to the discontinued HRD scores. Thus, we chose the median value of the candidate HRD scores, 38, as the threshold for positive HRD. Considering the good correlation between Pair Model and Single Model, the validation in Cohort II evaluated by the Single Model supported the shared threshold in these two HRD evaluation models.

In ovarian cancer, carrying *BRCA1/2* mutations, earlier stage, and lower level of residual tumor after surgery have been proven as predictors of better prognosis for patients receiving platinum-contained chemotherapy [20, 21]. However, in our Cohort II, patients with pathogenic *BRCA1/2* mutations didn’t show longer PFS and both cancer stage and the level of residual tumor were not significant factors in the Cox multivariate analysis. One possible explanation was the limited cohort size. While HRD status defined with the threshold of 38 successfully distinguished good responders regardless of *BRCA* status. Furthermore, over 80% (31/38) of HRD-positive and *BRCA*-intact patients were sensitive to platinum-based chemotherapy. In contrast, about 60% (12/19) of HRD-negative and *BRCA*-intact patients were still Pt-sensitive which indicated other response mechanisms. Therefore, the subsequent treatment strategies for recurrent Pt-sensitive patients remained debatable considering the multiple response mechanisms [22].

As reported in a previous pan-cancer study, the biallelic pathogenic alterations of *BRCA1/2* accounted for 68.7% of all cases harboring *BRCA1/2* mutations, which increased to 89.9% in *BRCA-*associated cancers [23]. Similarly, Lai et. al reported the fraction of *BRCA1/2* biallelic alterations as 94.0% and 84.7% in TCGA ovarian and breast cancer cohorts, respectively [24]. In our real-world Cohort III, *BRCA1/2* biallelic pathogenic alterations occurred in 73.4% (58/79) of all *BRCA1/2*-mutant cases which were all identified in *BRCA-*associated cancers. In addition, biallelic *BRCA1/2* and other HRR-related gene alterations were strongly associated with genome-wide LOH (gLOH) [23–25]. Worth noting, they all used gLOH in the evaluation of HRD status, rather than a combinatorial probe involving LOH, TAI, and LST, which was used in this study (the segment copy number variant information for HRD score evaluation was provided in Supplementary Table S8). But their findings still supported our observation that the HRD-positive subgroup contained a higher percentage of HRR gene deficiency than the HRD-negative subgroup (Fig. 5D).

Our GeneseeqPrime HRD panel is an integrated NGS panel that could evaluate HRD score and target 425 cancer-related genes including but not limited to *BRCA1/2* and other genes in the HRR pathway. Thus, it gave us the opportunity to investigate other potential mechanisms that could cause HRD besides *BRCA1/2* deficiency. *TP53* alteration has been identified as an early and critical pathogenic event in HGSOC and nearly 96% of them carried somatic *TP53* mutations [26]. Previous studies demonstrated the association between chromosomal instability and *TP53* mutations due to its role in controlling cell cycle checkpoints, DNA repair, and apoptosis [27]. Similarly, a study in high-grade endometrial carcinomas reported that *TP53* variants were more often present in HRD tumors than HR-proficient ones (100% vs. 41%; *p* = 0.019) but the HR status was determined by the quantification of RAD51-containing ionizing radiation-induced foci [28]. The overall frequency of *TP53* mutation was 93% and 79% in our Cohort II and ovarian cancer subgroup in Cohort III, respectively. Cohort II was comprised of only HGSOC patients, but Cohort III included all types of ovarian cancer, 26% (53/201) of which were diagnosed as HGSOC. Concurrent alteration analysis revealed higher *TP53* mutated frequency in HRD-positive patients in both Cohort II and ovarian subgroup in Cohort III.

*PTK2* (protein tyrosine kinase 2), a cytoplasmic protein tyrosine kinase, was reported to favor tumor progression and overexpressed in several advanced-stage solid cancers [29–31]. In our cohorts, *PTK2* amplification was only detected in HRD-positive ovarian patients. However, whether *PTK2* amplification promoted genome instability or resulted from HRD remained to be studied. In addition, we reported the enrichment of *NCOR1* loss in HRD-positive ovarian patients. It encodes a transcription factor and was identified as a prognostic biomarker of tamoxifen treatment in breast cancer [32, 33]. However, it was less investigated in ovarian cancer and the mechanism of association with a high HRD score was still unknown.

## Conclusions

In conclusion, we established two HRD score evaluation pipelines, Pair and Single Models, which showed high-level consistency and shared the same threshold of positive HRD. The HGSOC cohort with platinum-based chemotherapy survival data confirmed the role of HRD detection in identifying good responders. Pan-cancer HRD molecular analysis suggested the application of HRD detection in a real-world setting to guide treatment decisions.

## Supplementary Information

**Additional file 1: Figure S1. **The comparison of panel-based HRD (GeneseeqPrime® HRD) and WGS-based HRD score results. The HRD scores of 49 patients from Cohort I were evaluated by the panel-based HRD pipeline and whole-genome sequencing (WGS). The correlation of HRD scores between (A) Pair Model and WGS or (B) Single Model and WGS is shown with *BRCA* status labeled as the legend.

**Additional file 2: Figure S2. **Survival analysis of Cohort II. The Kaplan-Meier progression-free survival (PFS) curves of HRD-positive (blue) and HRD-negative (red) patients in the BRCA-intact subgroup (A) and BRCA-deficient/monoallelic pathogenic subgroup (B). (C) The PFS KM curves of all Cohort II patients were presented based on *BRCA *status (intact: blue vs. deficient+monoallelic pathogenic: red).

**Additional file 3: Figure S3. **Concurrent mutation analysis of Cohort III. The concurrentmutation analysis shows the significantly differently enriched gene alterationsin all HRD-positive and HRD-negative patients in Cohort III. *, *p *<0.05; **, *p *< 0.01; ***, *p *< 0.001**.**

**Additional file 4: TableS1. **A list of 25 HRR genes covered in targeted NGS panel.

**Additional file 5: Table S2. **Clinical characteristics of Cohort I and Cohort III.

**Additional file 6: Table S3. **High concordance of HRD status evaluated by GeneseeqPrime HRD Pair Model vs. WGS-based approach.

**Additional file 7: Table S4. **High concordance of HRD status evaluated by GeneseeqPrime HRD Single Model vs. WGS-based approach.

**Additional file 8: Table S5. **Clinical and genomic features of 85 samples from Cohort II.

**Additional file 9: Table S6. **Compelete list of mutations detected in 85 samples from Cohort II.

**Additional file 10: Table S7. **Genomic features of 416 samples from Cohort III.

**Additional file 11: Table S8. **Segment copy number variant information for HRD score evaluation of samples from Cohort II (*N* = 85) and Cohort III (*N* = 416).

## Data Availability

All data generated or analyzed during this study are included in this published article and its supplementary information files.

## Abbreviations

HRD: Homologous recombination deficiency
DDR: DNA damage repair
MMR: Mismatch repair
NER: Nucleotide excision repair
BER: Base excision repair
SSBs: Single-strand breaks
DSBs: Double-strand breaks
HRR: Homologous recombination repair
NHEJ: Non-homologous end joining
LOH: Loss of heterozygosity
TAI: Telomeric allelic imbalance
LST: Large-scale state transitions
PARPi: Poly ADP ribose polymerase inhibitors
SNPs: Single nucleotide polymorphisms
CNAs: Copy number alterations
NGS: Next generation sequencing
HGSOC: High-grade serous ovarian cancer
Pt: Platinum
PFI: Platinum-free interval
PFS: Progression-free survival
