# Supplementary figures and images for: Homologous recombination deficiency in diverse cancer types and its correlation with platinum chemotherapy efficiency in ovarian cancer
Source: BMC Cancer. 2022 May 16;22:550. doi: 10.1186/s12885-022-09602-4 (PMC9109318; doi:10.1186/s12885-022-09602-4)

A BRCA status ● Non-Deficient ● Deficient

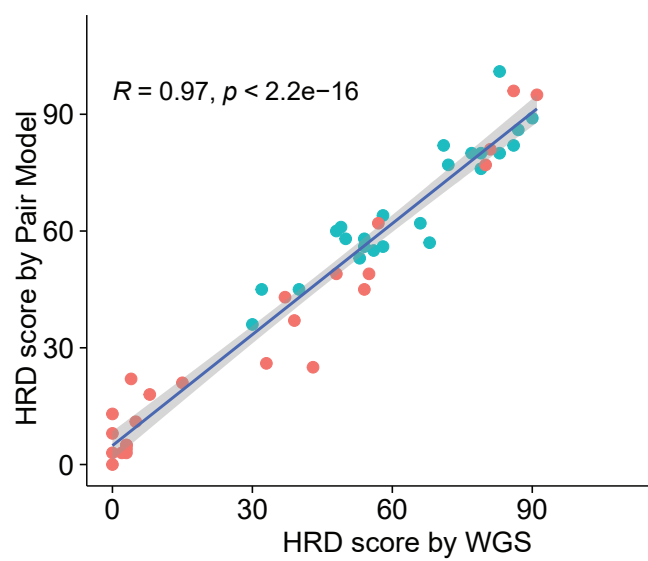

B BRCA status ● Non-Deficient ● Deficient

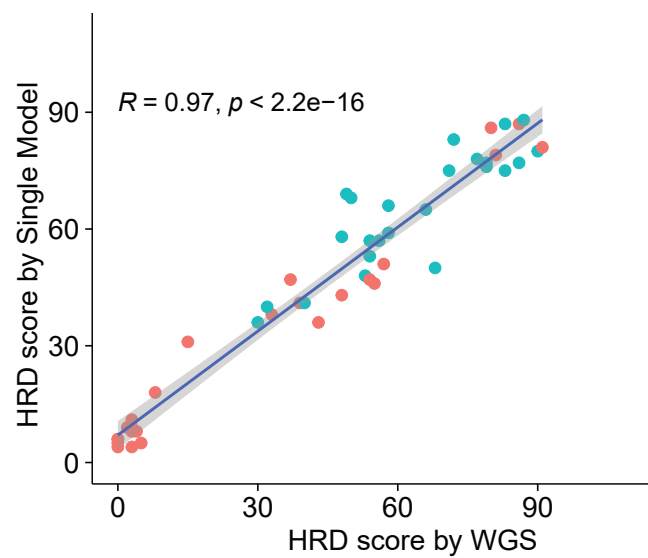

Supplement: Supplementary file 1 — Additional file 1: Figure S1. The comparison of panel-based HRD (GeneseeqPrime® HRD) and WGS-based HRD score results. The HRD scores of 49 patients from Cohort I were evaluated by the panel-based HRD pipeline and whole-genome sequencing (WGS). The correlation of HRD scores between (A) Pair Model and WGS or (B) Single Model and WGS is shown with BRCA status labeled as the legend. [file 12885_2022_9602_MOESM1_ESM.pdf]

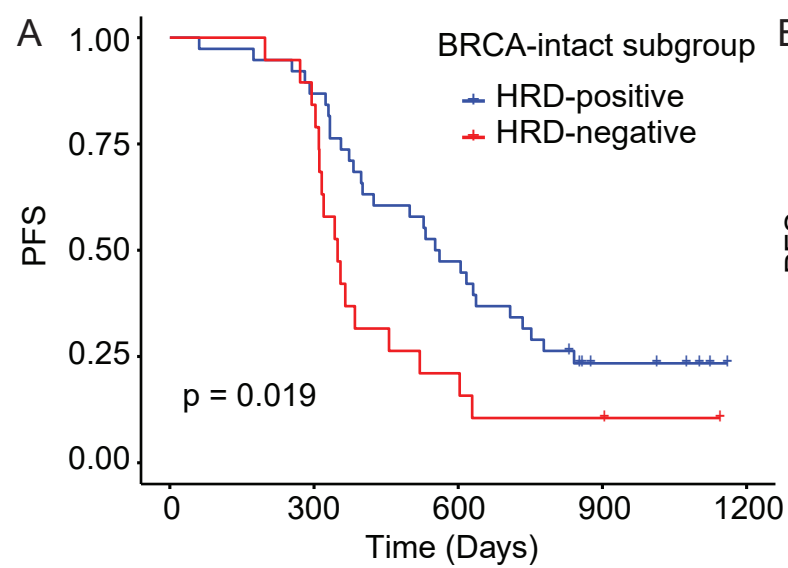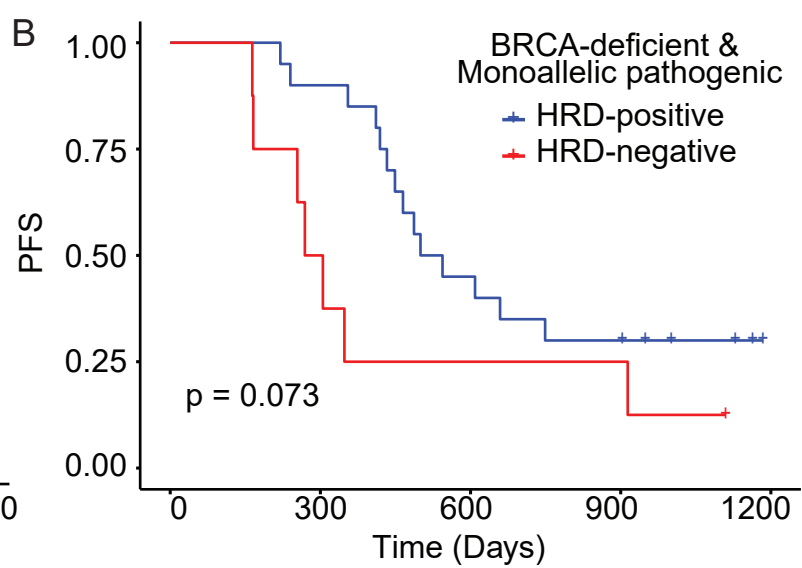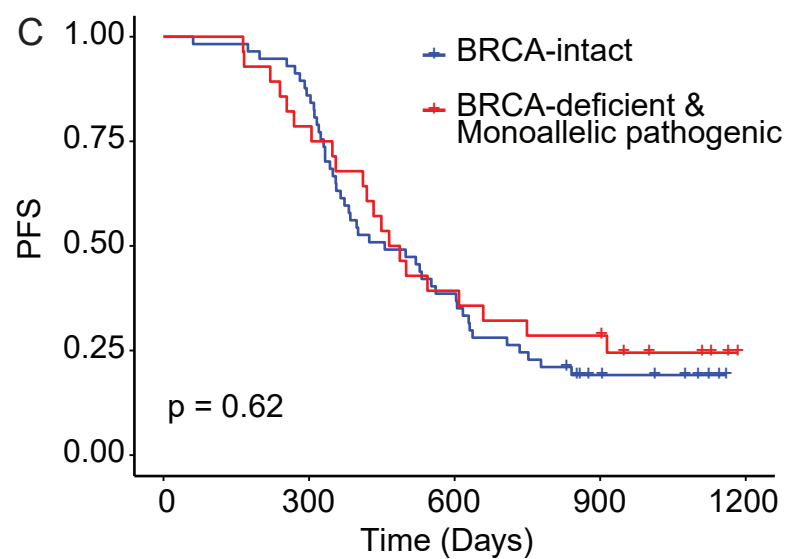

Supplement: Supplementary file 2 — Additional file 2: Figure S2. Survival analysis of Cohort II. The Kaplan-Meier progression-free survival (PFS) curves of HRD-positive (blue) and HRD-negative (red) patients in the BRCA-intact subgroup (A) and BRCA-deficient/monoallelic pathogenic subgroup (B). (C) The PFS KM curves of all Cohort II patients were presented based on BRCA status (intact: blue vs. deficient+monoallelic pathogenic: red). [file 12885_2022_9602_MOESM2_ESM.pdf]

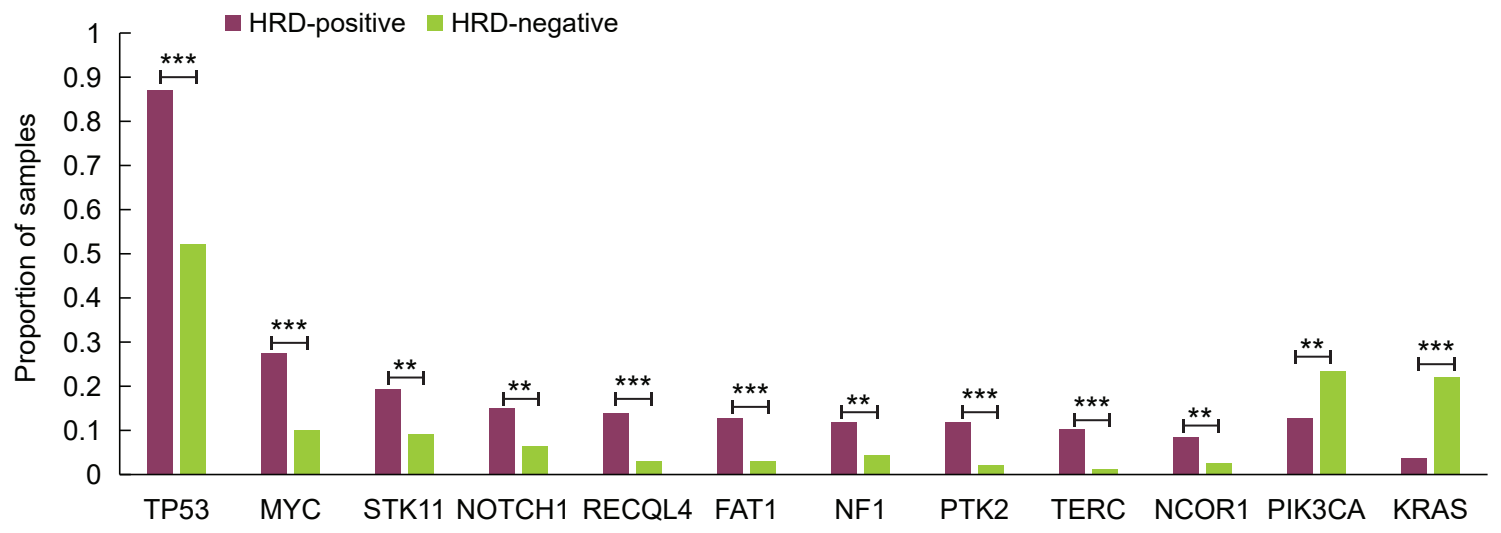

Supplement: Supplementary file 3 — Additional file 3: Figure S3. Concurrent mutation analysis of Cohort III. The concurrentmutation analysis shows the significantly differently enriched gene alterationsin all HRD-positive and HRD-negative patients in Cohort III. *, p <0.05; **, p < 0.01; ***, p < 0.001. [file 12885_2022_9602_MOESM3_ESM.pdf]
