# Supplementary material for: Homologous recombination deficiency in diverse cancer types and its correlation with platinum chemotherapy efficiency in ovarian cancer
Source: BMC Cancer. 2022 May 16;22:550. doi: 10.1186/s12885-022-09602-4 (PMC9109318; doi:10.1186/s12885-022-09602-4)
Supplement: Supplementary file 4 — Additional file 4: TableS1. A list of 25 HRR genes covered in targeted NGS panel. [file 12885_2022_9602_MOESM4_ESM.docx]

**Supplementary Table S1. A list of 25 HRR genes covered in targeted NGS panel**

| *ATM* | *ATR* | *BARD1* | *BLM* | *BRCA1* |
| --- | --- | --- | --- | --- |
| *BRCA2* | *BRIP1* | *CDK12* | *CHEK1* | *CHEK2* |
| *ERCC3* | *FANCA* | *FANCC* | *FANCD2* | *FANCI* |
| *FANCL* | *MRE11A* | *NBN* | *PALB2* | *RAD51* |
| *RAD51C* | *RAD51B* | *RAD51D* | *RAD54L* | *WRN* |
