# Supplementary material for: Homologous recombination deficiency in diverse cancer types and its correlation with platinum chemotherapy efficiency in ovarian cancer
Source: BMC Cancer. 2022 May 16;22:550. doi: 10.1186/s12885-022-09602-4 (PMC9109318; doi:10.1186/s12885-022-09602-4)
Supplement: Supplementary file 5 — Additional file 5: Table S2. Clinical characteristics of Cohort I and Cohort III. [file 12885_2022_9602_MOESM5_ESM.docx]

**Supplementary Table S2. Clinical characteristics of Cohort I and Cohort III**

| Characteristics | Cohort I: N (%) | Cohort III: N (%) |
| --- | --- | --- |
| Total number | 199 | 416 |
| Age: median (range) | 52 (20 - 83) | 52 (13 - 86) |
| Cancer type: Breast | 81 (40.7) | 120 (28.8) |
| Ovarian | 54 (27.1) | 201 (48.3) |
| Pancreatic | 34 (17.1) | 26 (6.3) |
| Prostate | 21 (10.6) | 17 (4.1) |
| Uterine | 9 (4.5) | 16 (3.8) |
| Other | 0 (0) | 36 (8.7) |
| Sex: Female | 151 (75.9) | 349 (83.9) |
| Male | 48 (24.1) | 67 (16.1) |
| *BRCA* status: Intact | 116 (58.3) | 337 (81.0) |
| Deficient | 64 (32.2) | 58 (13.9) |
| Monoallelic pathogenic | 19 (9.5) | 21 (5.1) |
