# Supplementary material for: Homologous recombination deficiency in diverse cancer types and its correlation with platinum chemotherapy efficiency in ovarian cancer
Source: BMC Cancer. 2022 May 16;22:550. doi: 10.1186/s12885-022-09602-4 (PMC9109318; doi:10.1186/s12885-022-09602-4)
Supplement: Supplementary file 6 — Additional file 6: Table S3. High concordance of HRD status evaluated by GeneseeqPrime HRD Pair Model vs. WGS-based approach. [file 12885_2022_9602_MOESM6_ESM.docx]

**Supplementary Table S3. High concordance of HRD status evaluated by GeneseeqPrime HRD Pair Model vs. WGS-based approach**

| HRD status | | Pair Model | |
| --- | --- | --- | --- |
|  |  | ≥ 38 | < 38 |
| WGS | ≥ 38 | 30 | 2 |
|  | < 38 | 2 | 15 |
| Cohen’s kappa = 0.81 | | | |

Note: Cohen’s kappa: 0.81 – 1.00 means almost perfect or perfect agreement
